# Do various types of prelacteal feeding (PLF) have different associations with breastfeeding duration in Indonesia? A cross-sectional study using Indonesia Demographic and Health Survey datasets

**DOI:** 10.1136/bmjgh-2023-014223

**Published:** 2024-06-10

**Authors:** Lhuri D Rahmartani, Maria A Quigley, Claire Carson

**Affiliations:** 1 Department of Epidemiology, Faculty of Public Health, Universitas Indonesia, Depok, Indonesia; 2 National Perinatal Epidemiology Unit, Nuffield Department of Population Health, University of Oxford, Oxford, UK

**Keywords:** Maternal health, Public Health, Epidemiology, Child health, Cross-sectional survey

## Abstract

**Introduction:**

Prelacteal feeding (PLF) is anything other than breastmilk given to newborns in the first few days of birth and/or before breastfeeding is established. PLF comes in many forms and is known as a challenge to optimal breastfeeding. Interestingly, both breastfeeding and PLF are common in Indonesia. This study investigated the association between PLF (any PLF, formula, honey, water and other milk) and breastfeeding duration.

**Methods:**

This study used Indonesia Demographic and Health Surveys data from 2002, 2007 and 2017. Sample sizes were 5558 (2007), 6268 (2007) and 6227 (2017) mothers whose last child was aged 0–23 months. We used Cox regression survival analysis to assess the association between PLF and breastfeeding duration, estimating hazard ratios (HR) for stopping earlier.

**Results:**

Overall PLF was prevalent (59%, 67% and 45% in 2002, 2007 and 2017, respectively), with formula being the most common (38%, 50% and 25%). No association between any PLF and breastfeeding duration in 2002 (HR 0.90 (95% CI 0.70 to 1.16)), but in 2007 and 2017, mothers who gave any PLF were more likely to stop breastfeeding earlier than those who did not (HR 1.33 (95% CI 1.11 to 1.61) and 1.47 (95% CI 1.28 to 1.69), respectively), especially in the first 6 months (HR 2.13 (95% CI 1.55 to 2.92) and 2.07 (95% CI 1.74 to 2.47), respectively). This association was more consistent for milk-based PLF. For example, HR in 2017 was 2.13 (95% CI 1.78 to 2.53) for prelacteal formula and 1.73 (95% CI 1.39 to 2.15) for other milk. The associations were inconsistent for the other PLF types. Prelacteal water showed no association while prelacteal honey showed some association with a longer breastfeeding duration in 2002 and 2007.

**Conclusion:**

The impact of PLF on breastfeeding duration varied by type. While this study supports current recommendations to avoid PLF unless medically indicated, the potential consequences of different PLF types on breastfeeding outcomes should be clearly communicated to healthcare providers and mothers. Further research should explore the reasons for the high PLF prevalence in this setting.

**What is already known on this topic**

- There are many types of prelacteal feeding (PLF) but there is little information on how each type of PLF affects breastfeeding.

**What this study adds**

- Some types of PLF, especially milk-based ones, are associated with a shorter duration of breastfeeding, while other types, such as prelacteal honey and water, did not have a strong or consistent association with breastfeeding duration.

**How this study might affect research, practice or policy**

- This study supports current breastfeeding/infant feeding guidelines and provides evidence on the impact of PLF on optimal breastfeeding which can be used by healthcare providers to inform mothers about their feeding decision.

## Introduction

Prelacteal feeding (PLF) refers to any food or drink other than breastmilk given to a newborn within the first few days after birth and/or before breastfeeding starts.^1–3^ PLF comes in many forms, such as infant formula, animal milk, water, tea, honey, juice and sugar water.^4^ The WHO/UNICEF recommends against PLF unless medically indicated.^5^ One of the reasons is because PLF potentially alters the gut microbiome which may increase the risk of neonatal mortality and morbidity.^6 7^ PLF may interfere with the positive feedback cycle in breastmilk production^3^ and has been shown to be associated with suboptimal breastfeeding practices.^6 8–14^ These practices include discarding of colostrum,^8^ delayed initiation of breastfeeding^6^ and reduced breastfeeding exclusivity and duration.^14^

A shorter duration of breastfeeding poses health risks for both mother and child, which includes reduced protection against maternal breast cancer and suboptimal nutrition for the child.^2 3^ Despite the growing literature that shows the associations between PLF and shorter breastfeeding duration and global recommendation to avoid PLF, more evidence is needed to further understand the extent of disruption PLF contributes to breastfeeding duration. As per WHO/UNICEF recommendation, medically indicated PLF does exist and is occasionally needed,^5 15^ often with the aim of improving breastfeeding.^16 17^ Furthermore, there are circumstances where PLF is given for other reasons, such as cultural or religious practices.^18^ Explaining to healthcare providers and mothers about potential risk and benefits of PLF becomes crucial as it will help them to be aware of the consequences and make an informed decision about infant feeding practices which will hopefully improve breastfeeding.

PLF practices and breastfeeding vary worldwide,^4 19^ hence perspectives from various settings are needed. Indonesia has a large population, about 275 million,^20^ that is under-represented in global infant feeding research. It also provides an interesting context to learn about PLF and breastfeeding continuation, because both breastfeeding and PLF are common in this country.^1^ The common practice of breastfeeding in Indonesia might be contributed by the influence of the major religions,^21 22^ and the endorsement from national programmes and regulations.^23^ The latest Indonesia Demographic and Health Survey (IDHS) in 2017 showed that the median breastfeeding duration was 21.8 months,^1^ which was close to the WHO recommendation of breastfeeding up to 2 years and beyond.^5^ Meanwhile, Indonesia’s PLF prevalence was about 45%,^1^ which is higher than the global average (roughly 33%).^24^

Furthermore, considering that PLF comes in different forms, it is important not to see PLF as one entity. There are also numerous types of PLF in Indonesia but the most common types of PLF are infant formula, other milk, honey and water.^25^ In addition to measuring the association between PLF and breastfeeding duration in Indonesia, this study also aims to see how this association differs among various types of PLF.

## Methods

This cross-sectional study used IDHS datasets.^26^ IDHS is a part of the DHS Programme, country-level representative household surveys that periodically collect data in the areas of population, health and nutrition from over 90 countries.^27^ IDHS has been conducted by the Indonesian government in collaboration with Demographic and Health Survey (DHS) experts that is, ICF International and with funding from the United States Agency for International Development (USAID). IDHS has been conducted typically every 3–5 years since 1987. For this study, we used the IDHS from the years 2002, 2007 and 2017 as those were the only available IDHS that collected data on the PLF variables and children’s age when their mother stopped breastfeeding. The IDHS data, data collection process and definition of the variables used here are described below and in more detail on the DHS website^27^ and IDHS reports.^1 28 29^

### Study population

**Figure 1:**
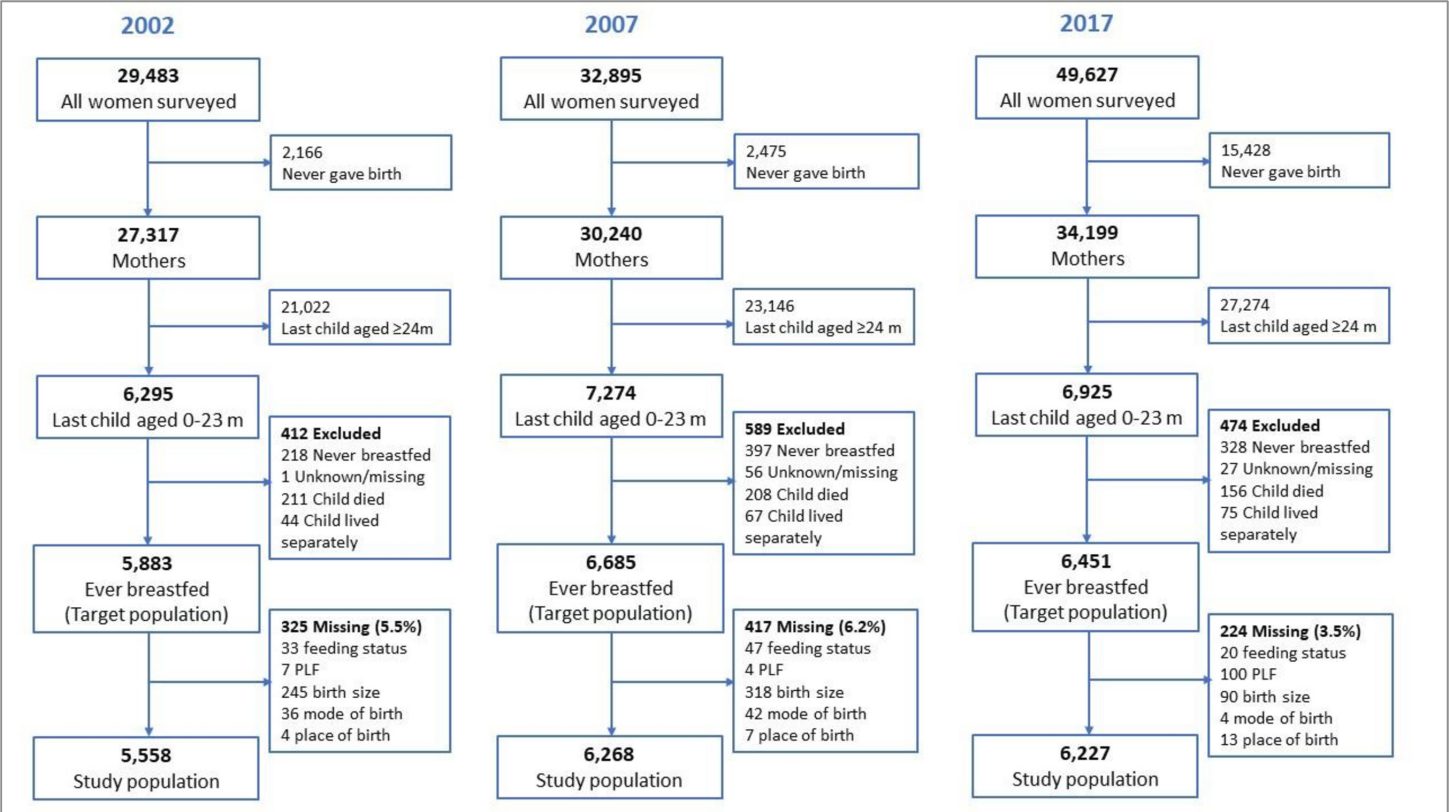
Selection of study population.

As shown in figure 1, the study population was mothers whose last child had ever been breastfed and was aged 0–23 months old at the time of the IDHS interview. This age range was selected by considering the WHO’s current recommendation which states that breastfeeding should continue for up to 2 years and beyond. Other guidelines may have a different age cut-off, but Indonesia follows the WHO recommendation.^23^ The child’s age in IDHS was measured in months, so children who were aged several weeks older than 23 months but had not yet had their second birthday would still be recorded as 23 months. Mothers whose children were never breastfed, died, were missing, lived separately and had missing values in any selected variables were excluded. The study population was 5558 in 2007, 6268 in 2007 and 6227 in 2017 (figure 1).

### Outcome variables

The outcome of interest is the timing of breastfeeding cessation that is, the child’s age at which the mother reported that she stopped breastfeeding her child. The age was recorded in months and the question only applied to the youngest (last) child at the time of the interview. For example, if the mother had two children or more at the time she was interviewed, only information about the youngest child was collected and analysed in this study. If the mother reported that she stopped breastfeeding before the interview, then the child’s age when she stopped breastfeeding was recorded as the timing of the event. Consequently, for mothers who were still breastfeeding at the time of interview, the data were censored at the age of the child when interview took place.

### Explanatory variables

Mothers were asked whether they gave any PLF (‘In the first 3 days after delivery, before your milk began flowing regularly, was (NAME) given anything to drink other than breastmilk?’). Those who answered YES were asked if they gave specific types such as formula milk and other milk). The explanatory variables analysed were any PLF, prelacteal formula, other milk, honey and water (all binary variables).

### Covariables (potential confounders)

The association between PLF and breastfeeding duration was adjusted for potential confounders. They were identified from the existing literature,^4 30–37^ and our previous study which was based on the 2017 IDHS^25^ and theoretically associated with both PLF and breastfeeding but not mediators. These were grouped as maternal demographic factors and child/birth-related factors. Maternal demographic factors were maternal age (15–19, 20–24, 25–29, 30–34 and ≥35), level of education (none/primary, secondary and higher), area of residence (urban and rural) and wealth quintile (Q1–Q5, with Q1 being the poorest and Q5 the wealthiest). The detailed calculation of wealth quintile, which is the per cent distribution of the de jure population by wealth quintiles and the Gini coefficient, can be found online (https://dhsprogram.com/Data/Guide-to-DHS-Statistics/Wealth_Quintiles.htm).^38^ Child/birth-related variables are child’s sex (male or female), birth order (whether this was first child or subsequent child), place of birth (home, public facility or private facility), mode of birth (vaginal or caesarean) and perceived birth size (smaller than average, average or larger than average). The detailed association between these variables and PLF can be found in our previous publication.^25^

### Statistical modelling

Each survey was analysed separately using the ‘svy’ commands in Stata V.15,^39^ which allow the use of probability weights to account for the complex sampling design and produce results that can be generalised to the target population. All proportions, hazard ratio (HR), and statistical tests are weighted while frequencies show true counts (unweighted). Survival analysis was used to compare the reported duration of breastfeeding in mothers who gave PLF (any PLF, formula, honey, water and other milk) and those who did not. The ‘event’ of interest was the cessation of breastfeeding. HR was obtained from Cox proportional hazards regression, where an HR larger than 1 is interpreted as the PLF group having a higher rate of breastfeeding cessation (ie, a shorter duration of breastfeeding) than those who did not give PLF.

Initially, the effect of PLF on breastfeeding duration was examined graphically using the Kaplan-Meier (KM) estimate of survival. The survival difference between PLF groups was formally tested using the log-rank test. A statistical test to check the proportional-hazards assumption based on Schoenfeld residuals was performed after fitting the Cox models. As there was evidence of an interaction with time in the Cox models (based on Schoenfeld residuals and visual inspection of the survival curves), an interaction with time was fitted, which resulted in HRs for three periods of observation, which were 0–6, 7–12 and 13–23 months. These cut-offs were based on clinical importance, where 0–6 is the duration of exclusive breastfeeding as recommended by WHO, 1 year is the continued breastfeeding duration recommended by other guidelines such as the American Academy of Pediatrics,^40^ and 2 years is the continued breastfeeding duration recommended by WHO.^3^ As PLF is given soon after birth, splitting observation time into these periods allowed clearer assessment of how PLF affected breastfeeding in the short and long term.

In the final multivariable model, the association between any PLF and breastfeeding duration was estimated, controlling for all potential confounders. Moreover, the association between each type of PLF was not only adjusted for maternal and child and birth-related variables but also for each of the other PLF types. For example, the association between prelacteal formula and breastfeeding duration was adjusted for potential confounders, prelacteal honey, water and other milk.

### Dealing with missing data

Over 98% of the target population in each survey year had complete data on the breastfeeding and PLF variables. A complete case analysis was preferred over multiple imputation or other strategies because the proportion of women with missing values for any of the key variables was considered small (3.5–6.2%) (figure 1).

### Sensitivity analysis

As this was a non-randomised study using a causal model to measure an association between two variables, sensitivity analysis was used to assess the robustness of the association to different assumptions that may affect the results, such as unobserved confounding.^41^ In the main analysis, we used the model with the largest population size for each survey and the exact same potential confounders throughout survey years. For each survey year, a sensitivity analysis was conducted by adjusting for three additional variables that were considered as potential confounders but were not available in all survey years and/or had missing values of >10% in at least one survey year. These potential confounders were maternal and child (MCH) book (missing >10% in 2002–2007), religion (only available in 2002–2007) and immediate skin-to-skin contact (only available in 2017). Religion may be associated with both PLF and breastfeeding. Possession of MCH book, also known as ‘antenatal card’ in the IDHS dataset, contained information about PLF and breastfeeding, thus having this book could influence decisions related to PLF and breastfeeding.

### Ethics review

Data were obtained from procedures and questionnaires that comply with standard DHS surveys (https://dhsprogram.com/What-We-Do/Protecting-the-Privacy-of-DHS-Survey-Respondents.cfm).^42^All protocols have been reviewed and approved by ICF Institutional Review Board (IRB) and an IRB in the host country, that is, Indonesia in this case.^42^ ICF IRB confirms that the survey conforms to the U.S. Department of Health and Human Services regulations for the protection of human subjects (45 CFR 46).^42^

### Patients and public involvement

Due to the nature of the study, patients and public involvement in the design, conduct, reporting or dissemination plans of our research was not applicable.

## Results

Online supplemental table S1 describes the characteristics of the study population. In 2002, 14.7% (95% CI 13.1% to 17.4%) of mothers whose child was aged 0–23 months had stopped breastfeeding at the time of interview. From 2007 to 2017, this proportion increased to 17.4% (95% CI 16.0% to 19.0%) and 20.5% (95% CI 19.3% to 21.8%), respectively. The proportion of women who had given any PLF was 59%, 67% and 45% in 2002, 2007 and 2017, respectively, with formula being the most common type (38%, 50% and 25%). The proportion of women who had given other milk increased across the surveys (from 0.3% to 14.0%) whereas the proportions who had given honey or water had decreased (honey from 12.8% to 3.5%; water from 10.4% to 4.9%) (online supplemental table S1).

10.1136/bmjgh-2023-014223.supp1 Supplementary data

Online supplemental table S1 also shows the 25th percentile for breastfeeding duration (this was a more useful measure than the median since 50% of mothers were still breastfeeding when their child was 23 months old). The duration at which 25% of mothers had stopped breastfeeding did not differ between those who gave any form of PLF and those who did not, at least in 2002 and 2007 (25th percentile 18 months in both groups). However, in all surveys, prelacteal formula was associated with a shorter breastfeeding duration. For example, in 2002, 25% of mothers who gave prelacteal formula stopped breastfeeding by 16 months compared with 20 months in the no prelacteal formula group. Conversely, prelacteal honey was associated with a longer breastfeeding duration in 2002 (25th percentile 21 months compared with 18 months in the no prelacteal honey group) and 2017 (25th percentile 20 months compared with 17 months).

**Figure 2:**
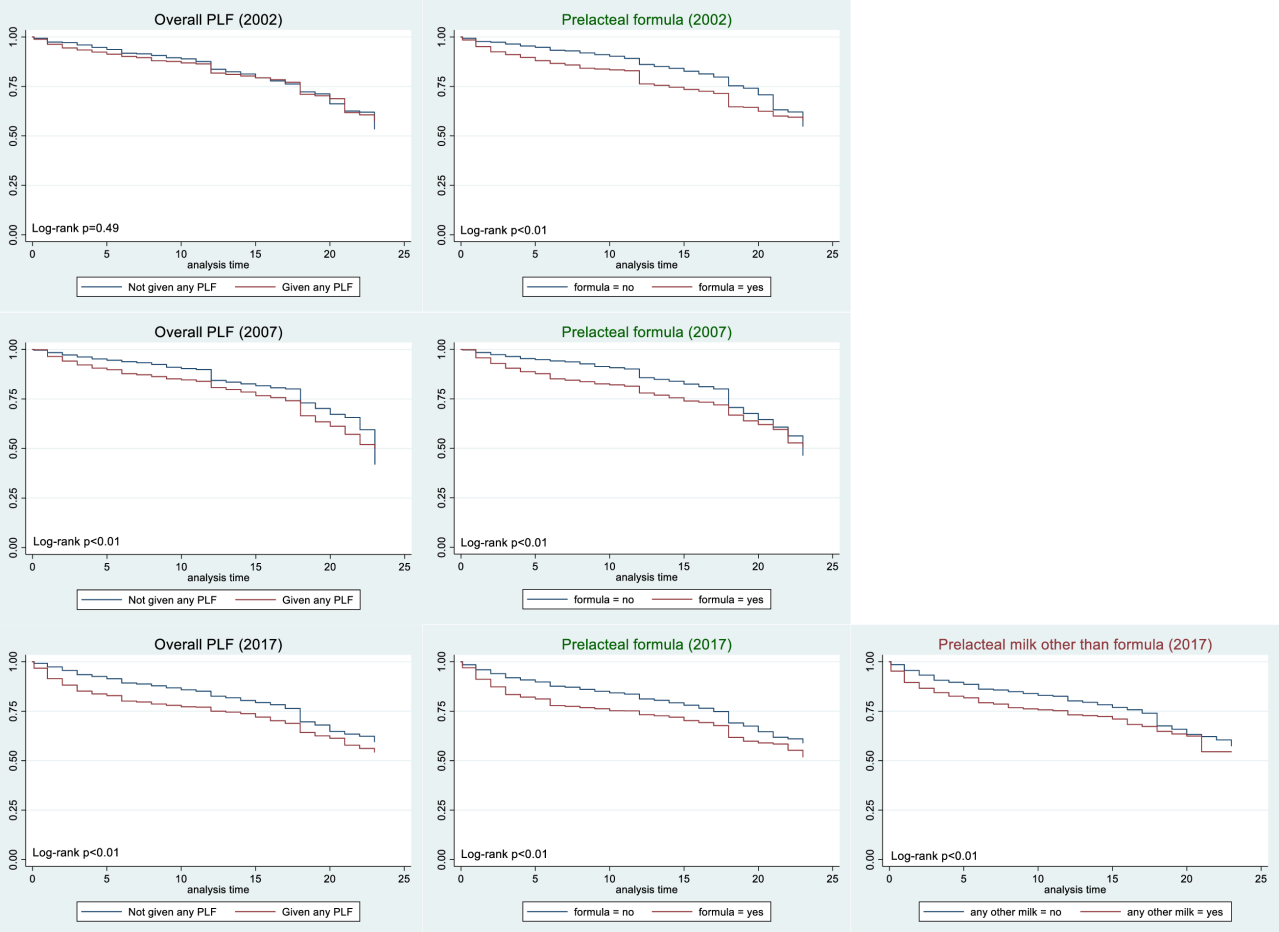
Kaplan-Meier curves for any PLF, prelacteal formula and other milk (2002–2017). PLF, prelacteal feeding.

**Figure 3:**
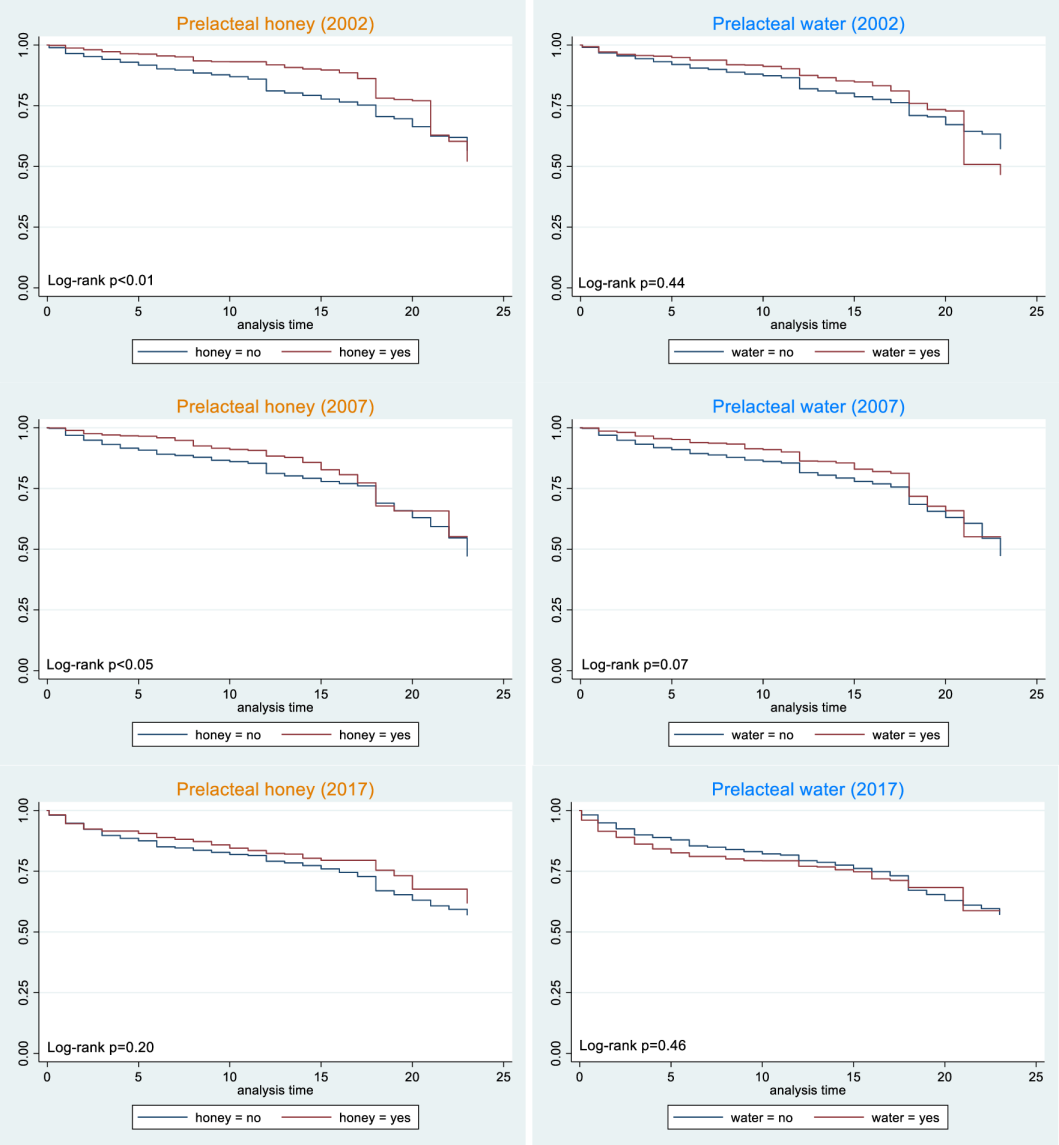
Kaplan-Meier curves for prelacteal honey and water.

Figures 2 and 3 show the KM curves for breastfeeding continuation rates and log-rank p values for all PLF types (ie, overall PLF and formula, other milk, honey and water) in all survey years. These were unadjusted and did not take potential confounders into account. Table 1 shows the HRs (overall and age-specific) for breastfeeding cessation for overall PLF and the four types of PLF. The direction of association, effect size, and statistical significance of the findings in the sensitivity analyses were not shown as they were relatively similar to those in the main analyses.

**Table 1:** Overall and time-specific HR and 95% CI of breastfeeding cessation for any PLF and each type for mothers whose child was ever breastfed and aged 0–23 months in the IDHS 2002, 2007 and 2017

Variable	2002		2007		2017	
	Crude HR	Adjusted HR	Crude HR	Adjusted HR	Crude HR	Adjusted HR
**Any PLF**						
HR 0–23 months	1.13 (0.88 to 1.46)	0.90 (0.70 to 1.16)	1.43 (1.18 to 1.72)	1.33 (1.11 to 1.61)	1.55 (1.36 to 1.77)	1.47 (1.28 to 1.69)
P value*	<0.001	<0.001	<0.001	<0.001	<0.001	<0.001
HR 0–6 months	1.43 (0.91 to 2.25)	1.12 (0.72 to 1.75)	2.32 (1.69 to 3.18)	2.13 (1.55 to 2.92)	2.17 (1.83 to 2.58)	2.07 (1.74 to 2.47)
HR 7–12 months	1.09 (0.68 to 1.74)	0.85 (0.55 to 1.33)	0.81 (0.60 to 1.10)	0.76 (0.56 to 1.03)	0.83 (0.61 to 1.13)	0.79 (0.58 to 1.08)
HR 13–23 months	0.85 (0.59 to 1.23)	0.70 (0.48 to 1.01)	1.23 (0.87 to 1.73)	1.18 (0.83 to 1.67)	0.98 (0.75 to 1.30)	0.91 (0.68 to 1.21)
**Formula**						
HR 0–23 months	1.71 (1.34 to 2.18)	1.07 (0.80 to 1.44)	1.53 (1.28 to 1.82)	1.19 (0.99 to 1.43)	1.56 (1.35 to 1.79)	1.58 (1.36 to 1.84)
P value*	<0.001	<0.001	<0.001	<0.001	<0.001	<0.001
HR 0–6 months	2.56 (1.68 to 3.90)	1.58 (0.99 to 2.50)	3.08 (2.31 to 4.11)	2.34 (1.73 to 3.16)	2.08 (1.75 to 2.46)	2.13 (1.78 to 2.53)
HR 7–12 months	1.67 (1.03 to 2.71)	1.03 (0.64 to 1.66)	0.92 (0.68 to 1.25)	0.72 (0.53 to 0.98)	0.73 (0.50 to 1.06)	0.73 (0.50 to 1.06)
HR 13–23 months	0.92 (0.62 to 1.36)	0.60 (0.39 to 0.91)	0.88 (0.63 to 1.24)	0.71 (0.50 to 1.00)	1.02 (0.74 to 1.39)	1.02 (0.73 to 1.41)
**Other milk**	The prevalence of ‘other milk’ was too low in these study years for meaningful analysis to be conducted.					
HR 0–23 months					1.37 (1.14 to 1.64)	1.46 (1.21 to 1.76)
P value*					<0.001	<0.001
HR 0–6 months					1.63 (1.32 to 2.02)	1.73 (1.39 to 2.15)
HR 7–12 months					1.16 (0.76 to 1.78)	1.32 (0.85 to 2.06)
HR 13–23 months					0.88 (0.58 to 1.34)	0.93 (0.60 to 1.46)
**Honey**						
HR 0–23 months	0.63 (0.45 to 0.90)	0.74 (0.55 to 1.01)	0.81 (0.60 to 1.08)	0.96 (0.71 to 1.30)	0.79 (0.55 to 1.14)	0.83 (0.57 to 1.22)
P value*	0.03	<0.001	<0.001	<0.001	0.89	<0.001
HR 0–6 months	0.45 (0.25 to 0.80)	0.55 (0.31 to 0.99)	0.39 (0.22 to 0.67)	0.47 (0.27 to 0.81)	0.79 (0.49 to 1.26)	0.82 (0.52 to 1.31)
HR 7–12 months	0.31 (0.16 to 0.59)	0.37 (0.19 to 0.70)	1.02 (0.61 to 1.71)	1.20 (0.71 to 2.02)	0.96 (0.47 to 1.99)	1.02 (0.49 to 2.13)
HR 13–23 months	1.20 (0.72 to 2.03)	1.33 (0.86 to 2.06)	1.28 (0.78 to 2.10)	1.53 (0.92 to 2.54)	0.67 (0.30 to 1.46)	0.71 (0.32 to 1.61)
**Water**						
HR 0–23 months	0.86 (0.60 to 1.22)	1.01 (0.72 to 1.42)	0.80 (0.59 to 1.08)	0.97 (0.72 to 1.30)	1.01 (0.75 to 1.37)	1.05 (0.76 to 1.45)
P value*	0.28	<0.001	0.05	<0.001	0.09	<0.001
HR 0–6 months	0.58 (0.29 to 1.20)	0.79 (0.39 to 1.62)	0.55 (0.34 to 0.91)	0.67 (0.41 to 1.09)	1.19 (0.83 to 1.70)	1.28 (0.89 to 1.84)
HR 7–12 months	0.68 (0.37 to 1.23)	0.84 (0.45 to 1.55)	0.94 (0.56 to 1.60)	1.10 (0.65 to 1.85)	0.64 (0.28 to 1.47)	0.67 (0.29 to 1.55)
HR 13–23 months	1.37 (0.80 to 2.35)	1.37 (0.83 to 2.24)	1.05 (0.61 to 1.79)	1.31 (0.78 to 2.22)	0.83 (0.43 to 1.60)	0.78 (0.39 to 1.55)

HR >1 (red font) indicates that PLF is associated with earlier cessation of any breastfeeding, HR <1 (blue font) indicates that PLF is associated with longer duration of breastfeeding.

*Indicates p value for interaction with time. Adjusted HRs were HRs after controlling for maternal demographic variables (age, education, wealth quintile, area of residence, regions) and child and birth related variables (sex, perceived birth size, place of birth, mode of birth) and PLF types.

HR, HR ratio; PLF, prelacteal feeding.

### Any PLF

There was no difference in breastfeeding duration between the PLF and no-PLF groups (p=0.49) in 2002 (figure 2). However, in 2007 and 2017, mothers who gave any PLF appeared to be more likely to stop breastfeeding earlier than those who did not. After adjusting for potential confounders, any PLF was found to be associated with shorter breastfeeding duration in 2007 and 2017 (table 1). In particular, mothers who gave any PLF were about 1.3–1.5 more likely to stop breastfeeding earlier compared with those who did not give any PLF. After fitting an interaction with time, this association was observed only in the first 6 months and was only statistically significant in 2007 (HR 2.13 (95% CI 1.55 to 2.92)) and 2017 (HR 2.07 (95% CI 1.74 to 2.47)) as shown in table 1.

### Prelacteal formula

Similar to any PLF, mothers who gave prelacteal formula were more likely to stop breastfeeding earlier than those who did not in all survey years (figure 2). However, after adjusting for potential confounders, this association was found to be statistically significant only in 2007 and 2017. Again, there was a significant interaction with time, with mothers who gave prelacteal formula being twice as likely to stop breastfeeding earlier in the first 6 months in 2007 and 2017 (table 1). In 2002, the HR was smaller (1.58) and the association was not statistically significant at the 5% level (table 1).

### Prelacteal milk other than formula

KM curves and HRs for prelacteal milk other than formula were only shown for the 2017 IDHS because the prevalence for this type of PLF was negligible in 2002 and 2007. Mothers who gave other milk as PLF were more likely to stop breastfeeding earlier than those who did not (figure 2). Again, the effect was stronger in the first 6 months, with mothers who gave other milk being 1.73 times more likely to stop breastfeeding earlier within the first 6 months (table 1).

### Prelacteal honey

In 2002, mothers who gave prelacteal honey were less likely to stop breastfeeding earlier than those who did not (figure 3). After adjusting for potential confounders (table 1), the association was not statistically significant for the period as a whole (0–23 months). After fitting an interaction with time, mothers who gave prelacteal honey in 2002 were less likely to stop breastfeeding earlier in the first 6 months (HR 0.55, 95% CI 0.31 to 0.99) and in the 7–12 months period (HR 0.37, 95% CI 0.19 to 0.70). However, the KM curves for prelacteal honey did not show the same patterns in 2007 and 2017 (figure 3). After adjusting for potential confounders and fitting an interaction with time, there was no statistically significant association between prelacteal honey and breastfeeding cessation except for being less likely to stop earlier in the first 6 months in 2007 (HR 0.47, 95% CI 0.27 to 0.81).

### Prelacteal water

The observed effects of prelacteal water on breastfeeding duration were the least consistent across survey years (figure 3). After adjusting for potential confounders and fitting an interaction with time (table 1), the association remained not statistically significant in most periods of all survey years. Prelacteal water was only statistically significant in the first 6 months in 2007, with crude HR 0.55 (95% CI 0.34 to 0.91).

## Discussion

### Summary

This study finds that half of the mothers were still breastfeeding when their child turned 23 months, which is in line with the current global recommendation.^2^ However, PLF was common, and giving any PLF was mostly associated with a shorter duration of breastfeeding. Generally, for any PLF and most types of PLF, the largest association was seen in the first 6 months. After this period, there was no evidence that cessation was associated with PLF. There were some differences in the association between each of the four types of PLF examined (formula, other milk, honey and water) and breastfeeding duration. Prelacteal formula and other milk were associated with a shorter breastfeeding duration in most survey years. However, prelacteal honey was associated with a longer breastfeeding duration in most survey years, whereas prelacteal water had no association in any survey year. These findings showed the importance of distinguishing among different types of PLF and instead of treating PLF as a single entity.

### Interpretation

#### Any PLF and breastfeeding duration

Results presented in this study indicate that mothers who gave any PLF to their child were more likely to stop breastfeeding earlier than those who did not. This is consistent with results from most studies of PLF and breastfeeding duration.^9–14^ The association was only apparent in the first 6 months, suggesting that PLF (particularly with formula) had the greatest influence in the weeks after breastfeeding initiation, but the importance of PLF as a predictor of breastfeeding wanes over time. It is likely overtaken by the impact of other factors such as introduction of complementary feeds (solids), mothers becoming pregnant again, or having other commitments that make breastfeeding difficult. Complementary feeds are expected to start at 6 months of age because breastmilk alone cannot fulfil their entire nutritional requirements.^2 3^ Although giving any PLF was associated with a shorter breastfeeding duration, this association appeared to differ across different types of PLF. This shows that the impact of PLF on breastfeeding is likely to be dependent on the substance given, which is also related to the amount, frequency, and, perhaps most importantly, the reason for giving it.

#### Prelacteal formula and breastfeeding duration

Prelacteal formula comprised the largest component of PLF types in all survey years, hence the pattern of association with breastfeeding duration was similar to that of any PLF. These findings suggest that prelacteal formula may affect breastfeeding duration through a mechanism that is different from other feeds such as prelacteal honey or water. Compared with these other feeds, formula has the closest features to breastmilk and is most likely given for nutritional purposes, rather than cultural or religious reasons. Hence, the chance of disrupting breastfeeding by making the baby feel full and decreasing the frequency of breastfeeding as well as stimulation for milk production^3^ is more plausible in this context. Consequently, supplementing with formula due to breastfeeding difficulties in the first few days can lead to more problems.^3 5^ In addition to disrupting the baby’s natural hunger and satiety, prelacteal formula is often given using bottles and teats which can affect the baby’s ability to suckle at the breast.^3 5^ Despite unclear causal evidence, the baby’s inability to suckle at the breast may contribute to maternal psychological stress, which may negatively affect the mother’s milk production.^43^ A combination of maternal stress and decreased frequency of baby’s suckling may create a vicious cycle of suboptimal breastfeeding.^3 5 43 44^

In the context of prelacteal formula, this study supports the current WHO guidelines^5 15^ that recommend avoidance of food or drink for newborns other than breastmilk, unless medically indicated. Our findings were also consistent with many studies from other countries which found that administration of formula in the early days of birth was associated with shorter breastfeeding duration,^9–11 13 14 45 46^ including a recent systematic review that looked at 48 prospective studies from countries in North America, East Asia and Pacific, South Asia, Latin America and Caribbean, Europe, North Africa and Sub-Saharan Africa.^14^ Our study, however, had a different finding from some experimental studies from the Czech Republic^16^ and the USA,^17^ which showed that a limited amount of formula feeding in the first days was found to have no adverse effects or instead improved breastfeeding duration. The study populations in the experimental studies were restricted to babies who lost at least 5% of their birth weight in the first few days. In clinical practice, this condition presents a dilemma for health practitioners, because medically indicated prelacteal formula is not always a clear-cut decision and can be affected by many factors, such as the mother’s or infant’s current medical conditions as well as the availability of infant formula and the supporting facilities.^15^ Moreover, a later study found that early limited feeding did not improve breastfeeding duration^47^ when it was introduced in a population with a high breastfeeding rate. These mixed findings show that there is a thin line between when PLF can be harmful and beneficial. However, WHO recommends that under normal conditions, a newborn does not need additional or substitute food/drink other than breastmilk in the first 2–4 days of life.^48^

Multiple factors contribute to breastfeeding success^49^ and the challenges to achieving it can vary among different populations and individuals.^50^ In Indonesia, some of the challenges include unethical formula marketing to women and health professionals and managing mothers’ own perception of poor milk supply.^51 52^ Qualitative studies in Jakarta reported that giving formula in the first days of birth contributed to difficulties in maintaining breastfeeding.^53–55^ Consequently, it is essential to be aware of the role of prelacteal formula as a starting point for breastfeeding disruption.

#### Prelacteal milk other than formula and breastfeeding duration

Our results suggest that prelacteal milk other than formula is associated with shorter breastfeeding duration although these results are more difficult to interpret. In Indonesia, ‘other milk’ can refer to a wide range of substances. Condensed milk, for instance, is widely consumed in Indonesia^56 57^ and is sometimes given as PLF.^58^ Other milk also possibly refers to plant-based milk, such as coconut milk, that is also reported to be given as PLF.^59^ There are also other milk products such as cow’s and buffalo’s milk, although their use as PLF is not specifically reported.^60^

#### Prelacteal honey and breastfeeding duration

To date, there is no published literature that comprehensively describes how prelacteal honey affects breastfeeding duration. In our study, prelacteal honey had an association with breastfeeding duration which was almost the opposite to that of prelacteal formula. Interestingly, in 2002 and 2007, giving prelacteal honey seemed to reduce the risk of early breastfeeding cessation. While the direction of the association was consistent across all survey years, the adjusted HR was not statistically significant in 2017. This is likely because the prevalence of prelacteal honey was much lower in 2017 than in earlier surveys, resulting in reduced statistical power.

It is unlikely that prelacteal honey promotes the continuation of breastfeeding; this finding probably reflects residual confounding, where the women who gave prelacteal honey were often the same individuals who breastfed for a longer time, without the association being causal. In our previous study, prelacteal honey was more prevalent among mothers with lower socioeconomic and education level,^25^ while in this study, breastfeeding was mostly longer among mothers with such backgrounds (online supplemental table S1). Although the association was adjusted for these variables, residual confounding may still be present.

Moreover, unlike formula, honey is often given for traditional or religious purposes, rather than as a designated nutritional replacement. For example in Indramayu, West Java, prelacteal honey was given with the intention to bless the baby and make him/her behave sweetly in the future and prevent the throat from drying up.^58^ More recently, the practice persists based on advice from parents, neighbours and older people in the family.^59^ It is often only a ritual that has been practised throughout generations and the amount of honey given may be so low that it has very little disruptive effect on breastfeeding.

Common religions in Indonesia (Islam, Christianity, Hinduism and Buddhism) are generally supportive towards breastfeeding,^21 22^ but both breastfeeding and honey are particularly valued in Islam,^61–63^ which constitutes 87% of all religions in Indonesia.^64^ In Islam’s holy book, Al-Quran, there are verses that specifically mention the importance of honey^62^ and the advice to breastfeed for 2 years.^61^ A number of studies report the use of honey as ‘tahnik’ or ‘tahneek’, a tradition practised by several groups of Muslims, which involves putting a sweet substance to a newborn baby’s mouth.^18 65 66^ While dates are used more often for this, honey can also sometimes be used for ‘tahnik’^55^ or other Islamic-related rituals to celebrate birth such as ‘aqiqah’.^59^

Despite no link between prelacteal honey and shorter breastfeeding duration, it is important to acknowledge other potential health risks of honey. To date, WHO still warns against giving honey to infants under 1-year old due to the risk of botulinum toxin.^67 68^ Although rare, the consequences can be life-threatening,^68–72^ thus avoiding unnecessary exposure remains the most sensible action.

#### Prelacteal water and breastfeeding duration

In our study, prelacteal water was found to have no effect on breastfeeding duration in any survey years. Although there are few studies looking at the effect of prelacteal water on breastfeeding duration, a study from Honduras also found no association between water PLF and breastfeeding duration.^73^ Moreover, some of the findings from a Cochrane review also showed that small amount of additional water or glucose water did not have any benefits on breastfeeding duration.^74^ These suggest that prelacteal water might not be detrimental to breastfeeding, but it might not be necessary either. Avoidance of prelacteal water remains sensible, as there is still a health risk beyond shorter breastfeeding duration, such as contamination, infection and gut microbiome alteration, especially in settings with restricted access to clean water.

### Strengths and limitations

To our knowledge, this is the first epidemiological study from Indonesia that investigates the association between different kinds of PLF and the duration of any breastfeeding. The main advantage of this study is that it is based on three national surveys and therefore is relatively large and representative at the country level. As described previously, the only quantitative study looking at PLF and any breastfeeding^75^ was conducted in a small region in Indonesia and cannot be generalised at the national level. To date, this is the first study from Indonesia and one of the very few studies that looked at the association between any breastfeeding and different types of PLF.

A potential limitation is that IDHS did not have sufficient details on some of the PLF variables such as ‘other milk’. The prevalence of other milk was very low in 2002 and 2007, and increased markedly in 2017, and the reasons for this are unclear. In addition, this study did not have information on the amount, frequency and reason why PLF was given. There were no data on the reason breastfeeding was stopped or data about breastfeeding intention and difficulties, which could have been more useful. Moreover, as with other studies on breastfeeding,^76^ recall bias was still likely to have occurred despite our effort to minimise it by limiting the period to less than 2 years. Finally, causal relationships cannot be established due to the cross-sectional study design.

### Policy implications

This study supports the current recommendation to avoid any kind of PLF whenever possible, but it also stresses the importance of understanding the potential impacts of each type of PLF. This is especially relevant in Indonesia or similar settings where mothers generally intend to breastfeed, hence this information could help them make an informed decision on how to feed their infants and seek support. This study also highlighted how common PLF is and the need to address it systematically. A potential public health strategy would include the provision of breastfeeding support starting from pregnancy to the early neonatal period, in conjunction with ensuring health workers are equipped to help manage early lactation problems.

## Conclusion

This study adds evidence of the negative association between PLF and the duration of any breastfeeding. Nevertheless, the negative association was observed for prelacteal formula and other milk, but not for honey and water. Giving a baby any PLF, especially milk-based feed, is more likely to be associated with a shorter breastfeeding duration, especially in the first 6 months after birth. Our study supports the WHO/UNICEF’s current guidelines that recommend against giving newborn infants food or drink other than breastmilk without medical indication while adding further clarification on how PLF potentially affects breastfeeding.

Although non-milk-based PLF generally had no association with breastfeeding duration, it does not mean that they are more acceptable (or ‘less harmful’) for babies, as this study did not look at other health risks, for example, infection and the potential alteration of the gut microbiome. This study discussed the need to collect data on the reason and quantity of feed given in the first few days of life. Giving a one-off prelacteal feed may have a negligible impact on breastfeeding duration, but with formula and other milk, the frequency might be higher (multiple times rather than a one-off). Nevertheless, more detail is required to properly understand what is happening. The potential consequences of different PLF types on breastfeeding outcomes should be communicated to healthcare providers and mothers. Further research should explore the reasons for the high levels of PLF in this setting.

## Data Availability

Data are available in a public, open access repository. IDHS dataset [26]. The DHS Program. Available Datasets: Indonesia (available from: https://dhsprogram.com/data/available-datasets.cfm. Data are available for research purposes on www.dhsprogram.com after registration.

## References

[1] National Population and Family Planning Board (BKKBN), Statistics Indonesia (BPS), Ministry of Health (Kemenkes), ICF . Indonesia Demographic and Health Survey 2017. Jakarta, Indonesia, 2018.

[2] WHO . Infant and young child feeding counselling: an integrated course, director’s guide. 2021. Available: http://apps.who.int/iris/handle/10665/343572

[3] WHO . Infant and young child feeding: model chapter for textbooks for medical students and Allied health professionals. 2009. Available: https://apps.who.int/iris/handle/10665/44117 23905206

[4] Neves PAR , Vaz JS , Ricardo LIC , et al . Disparities in early initiation of breast feeding and Prelacteal feeding: A study of Low- and middle-income countries. Paediatr Perinat Epidemiol 2022;36:741–9. 10.1111/ppe.12871 35253935

[5] WHO/UNICEF . Protecting, Promoting and Supporting Breastfeeding in Facilities Providing Maternity and Newbornservices: Implementing the Revised Baby-Friendly Hospital Initiative 2018. Geneva, 2018.

[6] Pérez-Escamilla R , Tomori C , Hernández-Cordero S , et al . Breastfeeding: crucially important, but increasingly challenged in a market-driven world. Lancet 2023;401:472–85. 10.1016/S0140-6736(22)01932-8 36764313

[7] Goldman AS . Modulation of the gastrointestinal tract of infants by human milk interfaces and interactions an evolutionary perspective. J Nutr 2000;130:426S–431S. 10.1093/jn/130.2.426S 10721920

[8] G/Slassie M , Azene ZN , Mulunesh A , et al . Delayed breast feeding initiation increases the odds of Colostrum avoidance among mothers in Northwest Ethiopia: a community-based cross-sectional study. Arch Public Health 2021;79:44. 10.1186/s13690-021-00571-x 33827691 PMC8028159

[9] Holmes AV , Auinger P , Howard CR . Combination feeding of breast milk and formula: evidence for shorter breast-feeding duration from the national health and nutrition examination survey. J Pediatr 2011;159:186–91. 10.1016/j.jpeds.2011.02.006 21429512

[10] Chantry CJ , Dewey KG , Peerson JM , et al . In-hospital formula use increases early Breastfeeding cessation among first-time mothers intending to exclusively Breastfeed. J Pediatr 2014;164:1339–45. 10.1016/j.jpeds.2013.12.035 24529621 PMC4120190

[11] Parry JE , Ip DKM , Chau PYK , et al . Predictors and consequences of in-hospital formula supplementation for healthy Breastfeeding newborns. J Hum Lact 2013;29:527–36. 10.1177/0890334412474719 23439865

[12] Sundaram ME , Labrique AB , Mehra S , et al . Early neonatal feeding is common and associated with subsequent Breastfeeding behavior in rural Bangladesh. J Nutr 2013;143:1161–7. 10.3945/jn.112.170803 23677862

[13] Nguyen TT , Withers M , Hajeebhoy N , et al . Infant formula feeding at birth is common and inversely associated with subsequent Breastfeeding behavior in Vietnam. J Nutr 2016;146:2102–8. 10.3945/jn.116.235077 27605404 PMC5037877

[14] Pérez‐Escamilla R , Hromi‐Fiedler A , Rhodes EC , et al . Impact of Prelacteal feeds and neonatal introduction of breast milk substitutes on Breastfeeding outcomes: a systematic review and meta-analysis. Maternal & Child Nutrition 2022;18:e13368. 10.1111/mcn.13368 35489107 PMC9113480

[15] WHO . Acceptable medical reasons for use of breast-milk substitutes. 2009. Available: https://www.who.int/nutrition/publications/infantfeeding/WHO_NMH_NHD_09.01_eng.pdf 24809113

[16] Straňák Z , Feyereislova S , Černá M , et al . Limited amount of formula may facilitate Breastfeeding: randomized, controlled trial to compare standard clinical practice versus limited supplemental feeding. PLoS ONE 2016;11:e0150053. 10.1371/journal.pone.0150053 26918700 PMC4769147

[17] Flaherman VJ , Aby J , Burgos AE , et al . Effect of early limited formula on duration and exclusivity of Breastfeeding in at-risk infants: an RCT. Pediatrics 2013;131:1059–65. 10.1542/peds.2012-2809 23669513 PMC3666109

[18] McKenna KM , Shankar RT . The practice of Prelacteal feeding to newborns among Hindu and Muslim families. J Midwifery Womens Health 2009;54:78–81. 10.1016/j.jmwh.2008.07.012 19114243

[19] Oakley L , Benova L , Macleod D , et al . Early Breastfeeding practices: descriptive analysis of recent demographic and health surveys. Matern Child Nutr 2018;14:e12535. 10.1111/mcn.12535 29034551 PMC5900960

[20] World Bank Group . Overview. Indonesia, 2023. Available: https://www.worldbank.org/en/country/indonesia/overview

[21] Papastavrou M , Genitsaridi SM , Komodiki E , et al . Breastfeeding in the course of history. JPNC 2015;2. 10.15406/jpnc.2015.02.00096

[22] Sugiri P , Ratnasari BC . Anjuran Menyusui dalam 6 Agama (Breastfeeding recommendation in 6 religions): Kumparan, 2019. Available: https://kumparan.com/kumparanmom/anjuran-menyusui-dalam-6-agama-1raSctVteqo/3

[23] Keputusan Menteri Kesehatan no.450 Th.2004 tentang Pemberian ASI Ekslusif . 2004.

[24] Neves PA , Armenta-Paulino N , Arroyave L , et al . Prelacteal feeding and its relationship with exclusive Breastfeeding and formula consumption among infants in Low- and middle-income countries. J Glob Health 2022;12:04104. 10.7189/jogh.12.04104 36560875 PMC9789363

[25] Rahmartani LD , Carson C , Quigley MA . Prevalence of Prelacteal feeding and associated risk factors in Indonesia: evidence from the 2017 Indonesia demographic health survey. PLoS One 2020;15:e0243097. 10.1371/journal.pone.0243097 33270720 PMC7714248

[26] The DHS Program . Datasets: Indonesia, Available: https://dhsprogram.com/data/available-datasets.cfm

[27] ICF International . DHS Overview: ICF International, Available: https://dhsprogram.com/What-We-Do/Survey-Types/DHS.cfm

[28] Badan Pusat Statistik-Statistics Indonesia - BPS, ORC Macro . Indonesia Demographic and Health Survey 2002-2003. Calverton, Maryland, USA: BPS and ORC Macro, 2003.

[29] Statistics Indonesia (Badan Pusat Statistik—BPS) . Macro International. Indonesia Demographic and Health Survey. Calverton, Maryland, USA: BPS and Macro International 2008, 2007.

[30] Peven K , Purssell E , Taylor C , et al . Breastfeeding support in low and middle-income countries: secondary analysis of national survey data. Midwifery 2020;82. 10.1016/j.midw.2019.102601 31935650

[31] Berde AS , Ozcebe H . Risk factors for Prelacteal feeding in sub-Saharan Africa: a Multilevel analysis of population data from twenty-two countries. Public Health Nutr 2017;20:1953–62. 10.1017/S1368980017000659 28443524 PMC10261358

[32] Birhan TY , Birhan NA , Alene M . Pooled prevalence and determinants of Prelacteal feeding practice in Eastern Africa evidence from demographic and health survey data: a Multilevel study. Risk Manag Healthc Policy 2021;14:1085–95. 10.2147/RMHP.S297564 33758561 PMC7979327

[33] Bergamaschi N , Oakley L , Benova L . Is childbirth location associated with higher rates of favourable early Breastfeeding practices in sub-Saharan Africa J Glob Health 2019;9:010417. 10.7189/jogh.09.010417 30774943 PMC6368939

[34] Teshale AB , Worku MG , Tessema ZT , et al . Prelacteal feeding practice and its associated factors among mothers having children less than 2 years of age in East Africa: a Multilevel analysis of the recent demographic and health surveys. Int Breastfeed J 2021;16:68. 10.1186/s13006-021-00414-z 34496922 PMC8424961

[35] Boccolini C , Perez-Escamilla R . Risk factors for Prelacteal feedings in seven Latin America and Caribbean countries. Ann Nutr Metab 2013;63:167.

[36] Boccolini CS , Pérez-Escamilla R , Giugliani ERJ , et al . Inequities in milk-based Prelacteal feedings in Latin America and the Caribbean: the role of cesarean section delivery. J Hum Lact 2015;31:89–98. 10.1177/0890334414559074 25421875

[37] Benedict RK , Craig HC , Torlesse H , et al . Effectiveness of programmes and interventions to support optimal Breastfeeding among children 0-23 months, South Asia: a Scoping review. Maternal & Child Nutrition 2018;14:e12697. 10.1111/mcn.12697 30499251 PMC6519148

[38] Croft TN , Marshall AMJ , Allen CK . Guide to DHS Statistics DHS-7 Rockville. Maryland, USA: ICF, 2018. Available: https://www.dhsprogram.com/pubs/pdf/DHSG1/Guide_to_DHS_Statistics_DHS-7.pdf

[39] StataCorp . Inventorstata statistical software: release 15. 2017.

[40] Eidelman AI , Schanler RJ , Johnston M , et al . Breastfeeding and the use of human milk. Pediatrics 2012;129:e827–41. 10.1542/peds.2011-3552 22371471

[41] Liu W , Kuramoto SJ , Stuart EA . An introduction to sensitivity analysis for unobserved confounding in Nonexperimental prevention research. Prev Sci 2013;14:570–80. 10.1007/s11121-012-0339-5 23408282 PMC3800481

[42] The DHS Program . Protecting the privacy of DHS respondents, Available: https://dhsprogram.com/What-We-Do/Protecting-the-Privacy-of-DHS-Survey-Respondents.cfm

[43] Dewey KG . Maternal and fetal stress are associated with impaired Lactogenesis in humans. The Journal of Nutrition 2001;131:3012S–3015S. 10.1093/jn/131.11.3012S 11694638

[44] Winberg J . Mother and newborn baby: mutual regulation of physiology and behavior--a selective review. Developmental Psychobiology 2005;47:217–29. 10.1002/dev.20094 16252290

[45] Petrova A , Hegyi T , Mehta R . Maternal race/Ethnicity and one-month exclusive Breastfeeding in association with the in-hospital feeding modality. Breastfeeding Medicine 2007;2:92–8. 10.1089/bfm.2006.0030 17661580

[46] Bolton TA , Chow T , Benton PA , et al . Characteristics associated with longer Breastfeeding duration: an analysis of a peer counseling support program. J Hum Lact 2009;25:18–27. 10.1177/0890334408325985 18971503

[47] Flaherman VJ , Cabana MD , McCulloch CE , et al . Effect of early limited formula on Breastfeeding duration in the first year of life: A randomized clinical trial. JAMA Pediatr 2019;173:729–35. 10.1001/jamapediatrics.2019.1424 31157878 PMC6547125

[48] WHO/UNICEF . Protecting, Promoting and Supporting Breastfeeding: The Special Role of Maternity Services–a Joint WHO/UNICEF Statement. Geneva, 1989. 1972080

[49] Victora CG , Bahl R , Barros AJD , et al . Breastfeeding in the 21st century: epidemiology, mechanisms, and lifelong effect. Lancet 2016;387:475–90. 10.1016/S0140-6736(15)01024-7 26869575

[50] Kavle JA , LaCroix E , Dau H , et al . Addressing barriers to exclusive breast-feeding in Low- and middle-income countries: a systematic review and programmatic implications. Public Health Nutr 2017;20:3120–34. 10.1017/S1368980017002531 28965508 PMC10262277

[51] Flaherman VJ , Chan S , Desai R , et al . Barriers to exclusive breast-feeding in Indonesian hospitals: a qualitative study of early infant feeding practices. Public Health Nutr 2018;21:2689–97. 10.1017/S1368980018001453 29973298 PMC10260846

[52] Susiloretni KA , Hadi H , Prabandari YS , et al . What works to improve duration of exclusive Breastfeeding: lessons from the exclusive Breastfeeding promotion program in rural Indonesia. Matern Child Health J 2015;19:1515–25. 10.1007/s10995-014-1656-z 25487415

[53] Fikawati S , Syafiq A . Penyebab Keberhasilan Dan Kegagalan Praktik Pemberian ASI Eksklusif (causes of successful and failed exclusive Breastfeeding). Jurnal Kesehatan Masyarakat Nasional (National Public Health Journal) 2009;4:120–31. 10.21109/kesmas.v4i3.184

[54] Novianti RA . Prelacteal feeding intake as one factor that led to the failure of exclusive Breastfeeding in textile industry workers in Jakarta. Indonesian Journal of Reproductive Health (Jurnal Kesehatan Reproduksi) 2014;5:23–6. 10.22435/kespro.v6i3.4737.137-144

[55] Minsarwati SY . Behaviors that hinder exclusive Breastfeeding practices amongst mothers in the area of Cibeber health care in 2009. Jurnal Kesehatan Reproduksi 2012;3.

[56] Den Hartog AP . Acceptance of milk products in Southeast Asia. In: Cwiertka KJ , Walraven BCA , eds. Asian Food: The Global and the Local. Honolulu: University of Hawaii Press, 2001: 40.

[57] Juffrie M , Sartika RAD , Sparringa RA , et al . Consumption patterns of sweetened condensed milk in the diet of young Indonesian children and its potential nutritional health consequences. Asia Pac J Clin Nutr 2020;29:16–26. 10.6133/apjcn.202003_29(1).0003 32229437

[58] Utomo B . Health and Social Dimensions of Infant Feeding: Lessons from Indramayu. West Java: ProQuest Dissertations Publishing, 1997.

[59] Hervilia D , Munifa D . Pandangan Sosial Budaya Terhadap ASI Eksklusif Di Wilayah Panarung Palangkaraya (social and cultural aspect toward exclusive Breastfeeding in Panarung Palangkaraya). IJHN 2016;3:63–70. 10.21776/ub.ijhn.2016.003.Suplemen.7

[60] Surono IS . Traditional Indonesian dairy foods. Asia Pac J Clin Nutr 2015;24 Suppl 1:S26–30. 10.6133/apjcn.2015.24.s1.05 26715081

[61] The Holy Qur’an 2:233 . Surah al-Baqarah verse 233.

[62] The holy Qur’An 16:69 . Surah an-NAHL verse 69.

[63] The Holy Qur’an 47:15 . Surah Muhammad verse 15.

[64] Statistics Indonesia (Badan Pusat Statistik—BPS) . Penduduk Menurut Wilayah dan Agama yang Dianut (Characteristic of population by province and religion), 2021. Available: https://sp2010.bps.go.id/index.php/site/tabel?tid=321&wid=0

[65] Asim M , Ahmed ZH , Hayward MD , et al . Prelacteal feeding practices in Pakistan: a mixed-methods study. Int Breastfeed J 2020;15:53. 10.1186/s13006-020-00295-8 32513203 PMC7278149

[66] Raheem RA , Binns CW , Chih HJ , et al . Determinants of the introduction of Prelacteal feeds in the Maldives. Breastfeed Med 2014;9:473–8. 10.1089/bfm.2014.0028 24964232

[67] WHO . Botulism. 2018. Available: https://www.who.int/news-room/fact-sheets/detail/botulism

[68] Abdulla CO , Ayubi A , Zulfiquer F , et al . Infant Botulism following honey ingestion. BMJ Case Rep 2012;2012. 10.1136/bcr.11.2011.5153 PMC3448763 22962382

[69] Godart V , Dan B , Mascart G , et al . Infant Botulism after honey exposure [Infant botulism after honey exposure]. Arch Pediatr 2014;21:628–31. 10.1016/j.arcped.2014.03.009 24768073

[70] Grant KA , McLauchlin J , Amar C . Infant Botulism: advice on avoiding feeding honey to babies and other possible risk factors. Community Pract 2013;86:44–6. 23914481

[71] Hoarau G , Pelloux I , Gayot A , et al . Two cases of type A infant Botulism in Grenoble, France: no honey for infants. Eur J Pediatr 2012;171:589–91. 10.1007/s00431-011-1649-5 22159905

[72] Wikström S , Holst E . Infant Botulism - why honey should be avoided for children up to one year. Lakartidningen 2017;114:ELMF. 28742188

[73] Pérez-Escamilla R , Segura-Millán S , Canahuati J , et al . Prelacteal feeds are negatively associated with breast-feeding outcomes in Honduras. J Nutr 1996;126:2765–73. 10.1093/jn/126.11.2765 8914947

[74] Smith HA , Becker GE . Early additional food and fluids for healthy Breastfed full-term infants. Cochrane Database Syst Rev 2016;2016:CD006462. 10.1002/14651858.CD006462.pub4 27574798 PMC8588276

[75] Sutayani DP . Correlation between Prelacteal Feeding and Breastfeeding Process in Work Area of Rowotengah Health Centre, District of Sumberbaru, Jember Regency. Jember: Jember University, 2012.

[76] Li R , Scanlon KS , Serdula MK . The validity and reliability of maternal recall of Breastfeeding practice. Nutr Rev 2005;63:103–10. 10.1111/j.1753-4887.2005.tb00128.x 15869124

