# Supplementary material for: Do various types of prelacteal feeding (PLF) have different associations with breastfeeding duration in Indonesia? A cross-sectional study using Indonesia Demographic and Health Survey datasets
Source: BMJ Glob Health. 2024 Jun 10;9(6):e014223. doi: 10.1136/bmjgh-2023-014223 (PMC11168184; doi:10.1136/bmjgh-2023-014223)
Supplement: Supplementary data [file bmjgh-2023-014223supp001.pdf]

Table S1. Characteristics of the study population of mothers whose child was ever breastfed and aged 0-23 months in the IDHS in 2002, 2007, and 2017

Variable	2002					2007					2017				
	Total		Stopped BF		25th perc.	Total		Stopped BF		25th perc.	Total		Stopped BF		25th perc.
	n	%	n	%	(months)	n	%	n	%	(months)	n	%	n	%	(months)
**Total**	**5,558**	**100.0**	**817**	**14.7**	18	**6,268**	**100.0**	**1,222**	**17.4**	18	**6,227**	**100.0**	**1,421**	**20.5**	17
**Child's age at interview**															
0-6 months	1,758	31.7	71	4.5	.	1,918	31.3	126	5.1	.	1,802	28.1	156	7.7	.
7-12 months	1,339	24.6	142	11.2	.	1,714	27.3	249	12.8	.	1,555	25.2	299	17.6	.
13-23 months	2,461	43.7	604	25.1	18	2,636	41.3	847	29.9	18	2,870	46.7	966	29.8	17
**PLF variables**															
**Any PLF**															
No	2,292	40.7	309	14.1	18	2,253	32.6	388	14.3	18	3,371	55.4	638	17.2	18
Yes	3,266	59.3	508	15.8	18	4,015	67.4	834	19.0	18	2,856	44.7	783	24.6	14
**Formula**															
No	3,505	62.5	439	12.6	20	3,425	50.5	567	14.8	18	4,639	74.7	954	18.6	18
Yes	2,053	37.5	378	19.4	16	2,843	49.5	655	20.1	16	1,588	25.3	467	26.3	12
**Other milk***															
No	5,538	99.7	813	15.2	18	6,245	99.7	1219	17.5	18	5,406	86.0	1,184	19.6	18
Yes	20	0.3	4	12.4	17	23	0.3	3	7.6	.	821	14.0	237	25.9	12
**Honey**															
No	4,798	87.3	721	15.6	18	5,565	89.1	1110	17.7	18	5,963	96.5	1,362	20.7	17
Yes	760	12.8	96	11.8	21	703	10.9	112	15.5	18	264	3.5	59	16.6	20
**Water**															
No	5,007	89.6	743	15.2	18	5,686	90.2	1132	17.7	18	5,898	95.1	1,345	20.5	17
Yes	551	10.4	74	14.6	21	582	9.8	90	15.2	18	329	4.9	76	21.5	16
**Maternal demographic**															
**Maternal age**															
15-19	358	6.9	42	17.5	12	354	5.6	44	11.1	18	296	4.4	55	16.2	18
20-24	1,457	26.8	228	15.8	18	1,552	25.4	317	17.2	18	1,220	20.0	292	22.2	16
25-29	1,619	28.4	247	16.7	18	1,820	28.2	383	20.3	16	1,702	26.8	407	22.1	15
30-34	1,199	19.7	176	12.8	21	1,436	22.1	286	18.0	18	1,589	25.8	336	19.4	18
>=35	925	18.2	124	13.3	20	1,106	18.7	192	14.7	19	1,420	23.0	331	19.3	18
**Level of education**															
None/primary	2,597	49.1	253	8.4	23	2,476	40.6	358	12.5	19	1,432	23.6	231	13.5	20
Secondary	2,541	44.1	455	20.1	16	3,243	50.7	693	19.3	18	3,556	59.4	800	20.9	17
Higher	420	6.9	109	31.7	8	549	8.7	171	29.7	7	1,239	17.0	390	28.9	8
**Wealth quintile**															
Q1 (least affluent)	1,708	21.0	180	10.3	20	1,751	20.5	240	13.1	18	1,637	19.8	279	14.6	18

Variable	2002					2007					2017				
	Total		Stopped BF		25th perc. (months)	Total		Stopped BF		25th perc. (months)	Total		Stopped BF		25th perc. (months)
	n	%	n	%		n	%	n	%		n	%	n	%	
Q2	1,065	18.9	117	8.0	22	1,233	18.5	212	13.8	18	1,252	20.4	254	16.0	18
Q3	963	20.2	126	13.5	20	1,153	21.0	205	14.9	18	1,151	20.0	260	20.4	17
Q4	897	20.3	163	16.0	20	1,094	20.7	258	19.4	18	1,126	20.7	291	22.7	16
Q5 (most affluent)	925	19.6	231	28.1	12	1,037	19.3	307	26.3	12	1,061	19.2	337	29.2	9
**Area of residence**															
Urban	2,342	47.9	460	20.7	15	2,433	42.0	608	21.8	14	3,060	48.5	847	25.0	13
Rural	3,216	52.1	357	10.1	20	3,835	58.1	614	14.3	18	3,167	51.5	574	16.3	18
**Region**															
Sumatera	1,631	22.9	230	14.8	18	1,946	22.7	403	20.3	23	1,654	22.9	353	19.5	17
Java	1,446	55.8	240	15.0	20	1,440	54.3	268	15.7	18	1,869	55.0	402	20.1	18
Bali-Nusa Tenggara	644	5.9	85	13.3	18	577	6.3	102	17.3	16	614	6.0	122	19.5	16
Kalimantan	768	6.6	96	13.2	22	628	6.1	125	19.7	15	535	5.6	141	22.4	16
Sulawesi	1,069	8.8	166	19.8	12	1,096	8.4	201	18.2	15	971	7.3	239	24.4	13
Maluku-Papua	NA	NA	NA	NA	NA	581	2.2	123	21.0	13	584	3.1	164	24.7	12
**Child and birth-related**															
**Sex of the child**															
Male	2,857	51.7	427	16.4	18	3,301	52.9	649	17.8	18	3,229	51.4	781	22.0	15
Female	2,701	48.3	390	13.8	19	2,967	47.1	573	17.1	18	2,998	48.6	640	18.9	18
**Birth order**															
First	1,828	32.3	330	21.0	15	2,063	35.3	479	20.7	16	1,981	32.6	556	25.8	12
Second/subsequent	3,730	67.7	487	12.3	20	4,205	64.7	743	15.6	18	4,246	67.5	865	17.9	18
**Perceived birth size**															
Larger	1,758	32.0	255	15.2	18	2,102	32.9	384	17.5	18	2,067	31.4	460	19.8	17
Average	3,045	55.4	444	15.3	18	3,227	53.5	657	17.2	18	3,384	57.6	756	20.2	17
Smaller	755	12.7	118	14.5	19	939	13.6	181	18.2	18	776	11.0	205	24.1	15
**Place of birth**															
Home birth	3326	55.7	367	10.5	21	3,488	49.0	561	14.1	18	1,291	15.9	277	18.2	18
Public facilities	617	10.0	115	20.8	13	911	11.6	195	21.1	16	2,419	34.4	490	18.2	18
Private facilities	1615	34.3	335	21.1	14	1,869	39.4	466	20.5	15	2,517	49.7	654	22.8	15
**Mode of birth**															
Vaginal	5,328	95.6	760	14.9	18	5,795	92.1	1099	17.0	18	5,083	81.1	1,084	19.3	18
Caesarean	230	4.4	57	21.6	15	473	7.9	123	22.6	12	1,144	18.9	337	25.6	12

*) Other milk in 2002 and 2007 was not included in the multivariable analysis due to low prevalence

PLF=prelacteal feeding, BF=breastfeeding, 25th perc.= 25th percentile of breastfeeding duration, NA=not available
